# Platinum-doublet chemotherapy for advanced gastroenteropancreatic neuroendocrine carcinoma: a systematic review and meta-analysis

**DOI:** 10.1007/s12672-022-00499-w

**Published:** 2022-05-30

**Authors:** Akihiro Ohmoto, Yu Fujiwara, Nobuyuki Horita, Kenji Nakano, Shunji Takahashi

**Affiliations:** 1 Department of Medical Oncology, The Cancer Institute Hospital of Japanese Foundation for Cancer Research, 3-8-31, Ariake, Koto-ku, Tokyo 1358550 Japan; 2 Department of Medicine, Icahn School of Medicine at Mount Sinai, Mount Sinai Beth Israel, 281 First Avenue, New York, NY 10003 USA; 3 Chemotherapy Center, Yokohama City University Hospital, 3-9 Fukuura, Kanazawa-ku, Yokohama, 236-0004 Japan

**Keywords:** Neuroendocrine carcinoma, Gastroenteropancreatic system, Platinum-doublet, Chemotherapy

## Abstract

**Background:**

Platinum-doublet chemotherapy has been conventionally used for patients with advanced gastroenteropancreatic (GEP) neuroendocrine carcinoma (NEC) but evidence of chemotherapy is based on studies with small sample sizes and remains scarce. Thus, we conducted a systematic review and meta-analysis to elucidate the efficacy of platinum-doublet chemotherapy for advanced GEP-NEC.

**Methods:**

We performed a database search in PubMed/MEDLINE and EMBASE. Eligible studies were prospective and retrospective studies documenting the efficacy of platinum plus etoposide (EP) and platinum plus irinotecan (IP) for advanced GEP-NEC. Overall response rate (ORR), median progression-free survival (PFS), and median overall survival (OS) were pooled and weighted using generic inverse variance in a random-effects meta-analysis model.

**Results:**

Nineteen studies including 1157 patients were identified. The ORR of the platinum-doublet regimen, EP, and IP was 49.1% (95% confidence interval [CI], 41.8–56.5), 44.4% (95% CI: 35.9–53.0), and 59.4% (95% CI: 48.0–70.8). The pooled median OS of the platinum-doublet regimen, EP, and IP was 12.9 months (95% CI:10.9–15.3), 12.9 months (95% CI: 10.8–15.4), and 12.9 months (95% CI: 6.0–27.8), and the pooled median PFS of the platinum-doublet regimen, EP, and IP was 5.4 months (95% CI: 4.5–6.4), 5.4 months (95% CI 4.5–6.5), and 4.0 months (95% CI: 1.4–11.7), respectively.

**Conclusion:**

Considerable response rate and survival time of the platinum-doublet regimen for advanced GEP-NEC were observed. IP and EP regimens can be reasonably applicable and these results provide a reference for oncologists in deciding the suitable regimen for patients with advanced GEP-NEC.

**Supplementary Information:**

The online version contains supplementary material available at 10.1007/s12672-022-00499-w.

## Introduction

Neuroendocrine carcinoma (NEC) is an aggressive pathological entity of neuroendocrine neoplasms (NENs) and is composed of small cell carcinoma and large cell NEC (LCNEC). According to the 2019 World Health Organization classification of tumors of the digestive system, poorly differentiated tumors with a high mitotic count (> 20 mitoses/10 high-power field) or high Ki-67 index (> 20%) are categorized as NEC [1]. The NEC occurs all over the body, and the most common primary site, except for the lung, is the gastroenteropancreatic (GEP) system. Owing to insufficient preclinical and clinical data, treatment strategies for extrapulmonary NEC are mainly based on those for small cell lung cancer (SCLC), which is based on morphological similarity. The most pervasive initial regimen for advanced cases is platinum-doublet chemotherapy, such as cisplatin (CDDP) plus etoposide (ETP), or CDDP plus irinotecan (CPT-11). To mitigate renal, gastrointestinal, and peripheral nerve toxicities, carboplatin (CBDCA) has become an alternative to CDDP in older and frail patients. The latest National Comprehensive Cancer Network (NCCN) guidelines for neuroendocrine tumors (NETs) are listed in parallel with CDDP/ETP, CBDCA/ETP, CDDP/CPT-11, and CBDCA/CPT-11 as platinum-doublet chemotherapy for unresectable or metastatic extrapulmonary NEC [2]. The selection of these regimens is chiefly based on toxicity profiles, patients’ performance status, and their preferences. However, only a few prospective trials have been conducted, and most of the available data were derived from small-scale retrospective studies. Consequently, the reported efficacy of platinum-doublet chemotherapy is heterogeneous among studies, and robust clinical evidence does not exist. To provide helpful information for daily practice, we aimed to conduct a systematic review and meta-analysis of available prospective and retrospective studies evaluating platinum-doublet regimens for GEP-NEC.

## Materials and methods

### Protocol registration

This systematic review and meta-analysis was performed based on the Preferred Reporting Items for Systematic Reviews and Meta-Analysis criteria. Prospective and retrospective studies that analyzed the efficacy of platinum-containing regimens in patients with advanced GEP-NEC were included in this analysis. The protocol was registered in the PROSPERO registry (CRD42021243614).

### Data sources and searches

Database searches were conducted using the PubMed/MEDLINE and Embase databases. The following keywords were used for literature retrieval: ((neuroendocrine carcinoma) OR (NEC) OR (neuroendocrine tumor) OR (neuroendocrine neoplasm) OR (small cell carcinoma)) AND (((platinum) and (etoposide)) or ((platinum) and (irinotecan)) or ((cisplatin) and (etoposide)) or ((carboplatin) and (etoposide)) or ((cisplatin) and (irinotecan)) or ((carboplatin) and (irinotecan)) or (platinum-based)). We searched these databases on July 07, 2021. Two authors (AO and YF) independently performed additional manual searches.

### Selection criteria

Eligible studies included randomized trials, single-arm trials, prospective observational studies, and retrospective studies on the efficacy of platinum-based chemotherapy for advanced NEC. Case reports and case series with a sample size of three or fewer cases were excluded because of insufficient evaluation of the efficacy of chemotherapy. All the eligible articles were written in English.

We selected advanced or inoperable patients pathologically diagnosed with NEC using the GEP system to conduct a compressive review of platinum-based chemotherapy for these populations. Reports of well-differentiated NETs were excluded.

Studies evaluating either a platinum plus ETP regimen (EP) or a platinum plus CPT-11 regimen (IP) were included in the analysis. Platinum-based regimens with different doses and densities were regarded the same because of the rarity of NEC. Reports evaluating chemotherapy in the neoadjuvant or adjuvant settings were excluded.

### Data extraction and risk of bias assessment

Two authors independently extracted data from eligible studies (AO and YF). Study characteristics included the name of the first author, title of each study, publication year, number of participants, sex, age, clinical stage, primary site, chemotherapy regimen, dosage and intensity of chemotherapy, hazard ratio (HR) of overall survival (OS) and progression-free survival (PFS), survival ratio of OS and PFS at a specific point, median OS and PFS with 95% confidence interval (95% CI), overall response rate (ORR), and complete response rate (CRR). Risk of bias was assessed using the 9-star Ottawa Newcastle Scale for non-randomized studies and the Cochrane risk of bias tool for randomized controlled trials.

### Data synthesis and statistical analysis

The pooled ORR, CRR, median PFS, and median OS were calculated using a random-effects meta-analysis model with the generic inverse variance method. Agresti-Coull adjustment was applied to estimate the standard error of the binomial proportion [3]. RevMan 5.4 was used for calculating these data [4]. The statistical significance threshold was set at p < 0.05. A two-tailed level of 0.10 and I^2^ < 50% were set for heterogeneity.

## Results

### Study selection

**Fig. 1:**
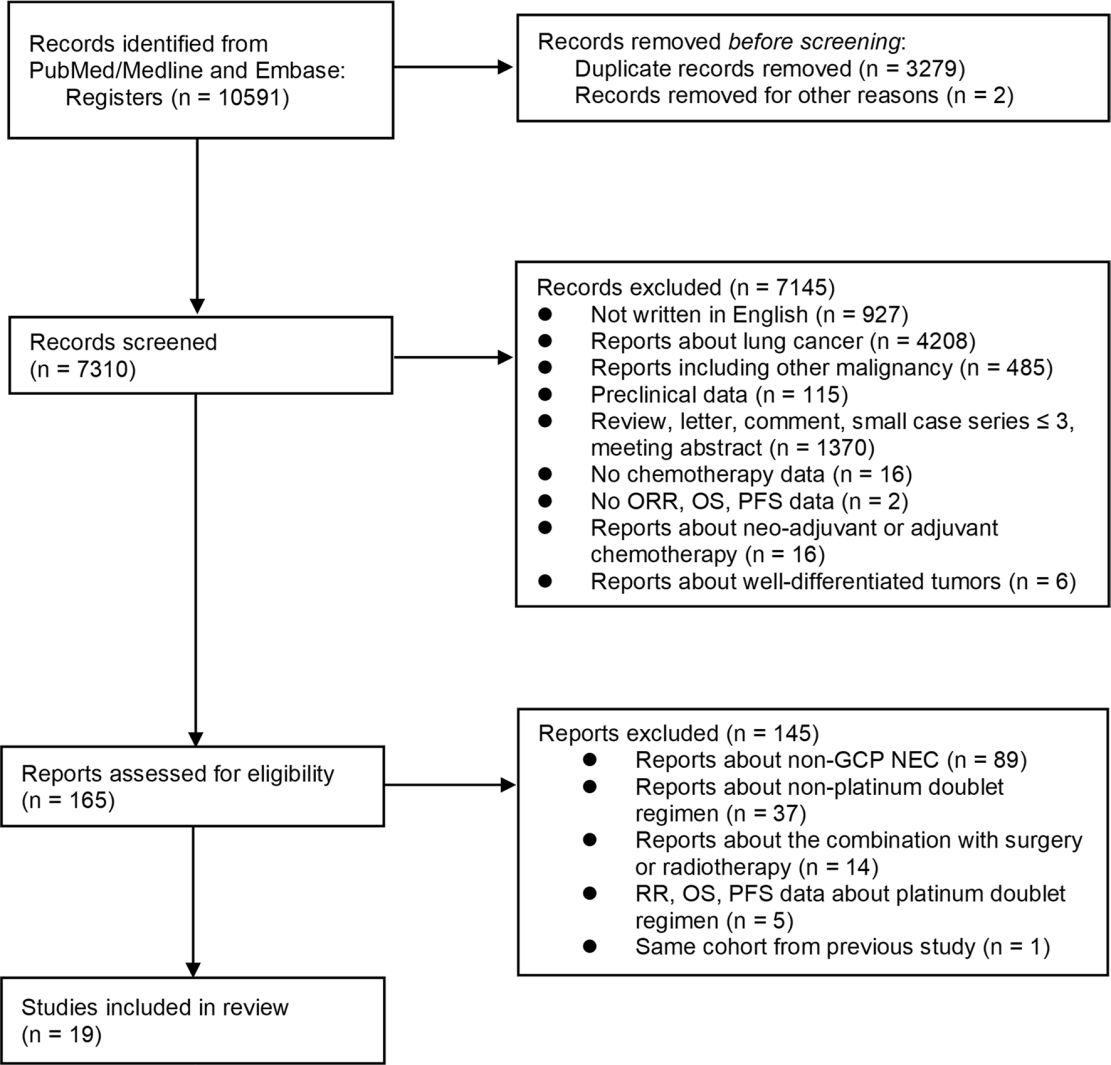
Flowchart of the systematic review process. On the basis of the initial screening and eligibility assessment, 19 studies with 1157 patients were identified

By searching the databases, 10,591 articles were found, 3279 articles were removed due to duplicates, and 7310 studies underwent further screening. Reports on lung cancer (n = 4208) were removed during this screening process, and 165 articles were potentially eligible after screening for the abstracts and titles. Of these studies, 146 reports were excluded, and 19 reports that included 1,157 patients were finally identified [5–23]. A flowchart of the study selection process is shown in Fig. 1.

### Study characteristic and treatment group description

Among the 19 studies, three directly compared the IP and EP regimens, of which one was a randomized phase II trial and the other two were retrospective studies [5, 6, 23]. The other 16 studies included one prospective study evaluating EP [15] and 15 retrospective single-arm studies, among which four studies analyzed IP and 11 studies evaluated EP. All the studies evaluated patients with advanced GEP-NEC who received systemic chemotherapy (IP or EP). Key study characteristics are summarized in Table 1. Among 430 patients whose primary organs were reported, the most common was the esophagus (29%), stomach (25%), and pancreas (22%). Information regarding the chemotherapy setting was available in 17 studies (Table 1). Among these studies, 14 included only chemo-naïve patients in the first-line setting. Regarding the remaining three studies, Zhang et al. evaluated the first-line treatment or adjuvant chemotherapy, Lu et al. included 15 patients in the first-line setting and one patient in the later-line, and Pulvirenti reported that 97% of included patients were treated with platinum-chemotherapy in the first-line setting [5, 9, 23]. 9-star Ottawa Newcastle Scale was used to evaluate the risk of bias of the included 18 studies, and the Cochrane risk bias assessment was used for one randomized phase II trial (Supplementary Table 1).

**Table 1:** Characteristics of selected studies

Authors [Reference]	Study design	Regimen	Treatment setting	Number of patients	Age (median)	Primary organ	Detailed primary organ
Zhang [5]	Randomized phase II	CDDP + ETP	Chemo-naïve or treated as adjuvant chemotherapy	33	NA	GEP (N = 58), unknown primary (N = 8)	Stomach (N = 20), Esophagus (N = 13), Colorectum (N = 11), Unknown primary (N = 8), Pancreas (N = 7), Duodenum (N = 4), Small intestine (N = 3)
		CDDP + CPT-11		33	NA		
Yamaguchi [6]	Retrospective	CDDP + ETP	Chemo-naïve	46	NA	GEP	Esophagus (N = 75), Stomach (N = 58), Pancreas (N = 29), Hepato-biliary tract (N = 23), Colorectum (N = 17), Small bowel (N = 4)
		CDDP + CPT-11		160	NA		
Sorbye [7]	Retrospective	CDDP + ETP	Chemo-naïve	129	NA	GEP, unknown primary	NA
		CBDCA + ETP		67	NA		
Okuma [8]	Retrospective	CDDP + CPT-11	Chemo-naïve	12	62	GEP	Esophagus (N = 12)
Lu [9]	Retrospective	CDDP + CPT-11	Chemo-naïve (N = 15) or treated (N = 1)	16	57	GEP (N = 15), unknown primary (N = 1)	Stomach (N = 8), Esophagus (N = 5), Small intestine (N = 1), Pancreas (N = 1), Unknown primary (N = 1)
Iwasa [10]	Retrospective	CDDP + ETP	Chemo-naïve	21	57	GEP	Pancreas (N = 10), Gallbladder (N = 8), Liver (N = 2), Ampulla of Vater (N = 1)
Okita [11]	Retrospective	CDDP + CPT-11	NA *	12	62	GEP	Stomach (N = 12)
Chin [12]	Retrospective	CDDP + CPT-11	Chemo-naïve	12	66	GEP	Esophagus (N = 12)
Yoon [13]	Retrospective	CDDP + ETP	Chemo-naïve	64	57	GEP	Gastrointestinal tract (N = 31), Hepatobiliary tract (N = 25), Abdominal lymph node (N = 8)
Patta [14]	Retrospective	CDDP + ETP	Chemo-naïve	8	64	GEP	Colorectum (N = 8)
Walter [15]	Prospective cohort	Platinum + ETP	Chemo-naïve	152	NA	GEP, unknown primary	NA
Brandi [16]	Retrospective	Platinum + ETP	Chemo-naïve	21	58	GEP (N = 16), unknown primary (N = 5)	Pancreas (N = 10), unknown primary (N = 5), stomach (N = 2), colon (N = 2), duodenum (N = 1), gallbladder (N = 1)
Heetfeld [17]	Retrospective	Platinum + ETP	Chemo-naïve	113	NA	GEP	NA
Bongiovanni [18]	Retrospective	Platinum + ETP	Chemo-naïve	20	60	GEP	Stomach (N = 8), pancreas (N = 7), colorectum (N = 5)
Hudson [19]	Retrospective	CBDCA + ETP	NA *	6	69	GEP	Esophagus (N = 6)
Sakamoto [20]	Retrospective	CBDCA + ETP	Chemo-naïve	4	66.5	GEP	Pancreas (N = 4)
Kim [21]	Retrospective	CDDP + ETP	Chemo-naïve	17	NA	GEP	NA
Gerard [22]	Retrospective	Platinum + ETP	Chemo-naïve	24	NA	GEP, unknown primary	NA
Pulvirenti [23]	Retrospective	Platinum + ETP	Chemo-naïve **	22	NA	GEP	Pancreas (N = 26)
		Platinum + CPT-11		4	NA		

*CDDP* cisplatin, *CPT-11* irinotecan, *ETP* etoposide, *GEP* gastroenteropancreatic, *NA* not available

^*^Although detailed treatment settings were not clearly documented in the article, the information from the literature suggests the 1st-line setting treatment

^**^97% of patients received platinum-based chemotherapy in the 1st-line setting

### Meta-analysis of response rate

**Fig. 2:**
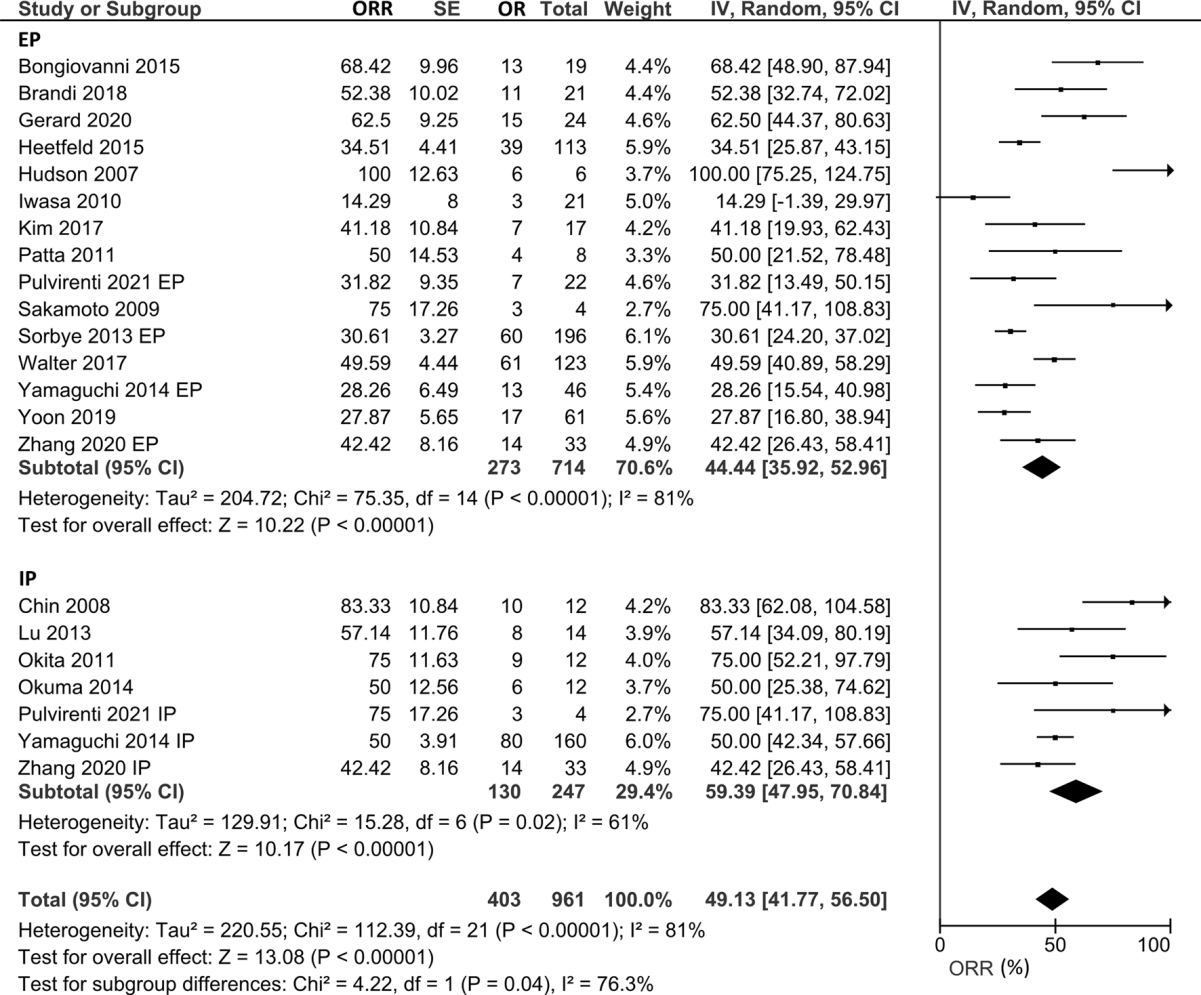
Pooled ORR in patients treated with platinum-doublet chemotherapy. 15 studies that evaluated patients treated with EP and seven studies that evaluated patients with IP were included in this analysis. *ORR* overall response rate, *EP* platinum plus etoposide, *IP* platinum plus irinotecan

**Fig. 3:**
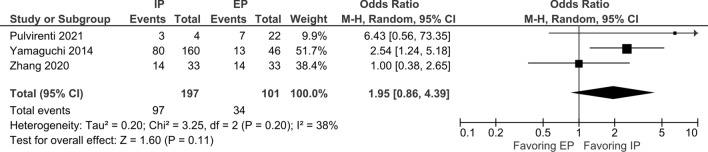
Odds ratio of ORR using EP as reference. Three studies that directly compared the ORRs of IP and EP were included in this analysis. Odds ratios were calculated using EP as a reference. Among them, one was a randomized phase II trial (5) and the other two were retrospective studies (6, 23). *EP* platinum plus etoposide, *IP* platinum plus irinotecan, *ORR* overall response rate

We evaluated ORR and CRR in patients with NEC who received EP and IP. For ORR, 15 studies that evaluated 987 patients treated with EP and seven studies that included 377 patients treated with IP were included. The pooled ORR in all patients who received the platinum-doublet regimen was 49.1% (95% CI 41.8–56.5%), and subgroup analysis for the EP and IP groups showed ORR of 44.4% (95% CI 35.9–53.0%) and 59.4% (95% CI 48.0–70.8%), respectively (Fig. 2). The CDDP/ETP (seven studies) and CBDCA/ETP (three studies) groups showed ORR of 31.0% (95% CI 24.3–7.6%) and 67.2% (95% CI 18.0–116.3%), respectively. For 370 patients in six studies who received CDDP/CPT-11, the pooled ORR was 58.1% (95% CI 46.1–70.0%) (Supplementary Fig. 1). Three studies compared the efficacy of EP and IP directly, and the odds ratio of ORR using EP as a reference was 1.95 (95% CI 0.86–4.39%, *p* = 0.11) (Fig. 3).

**Fig. 4:**
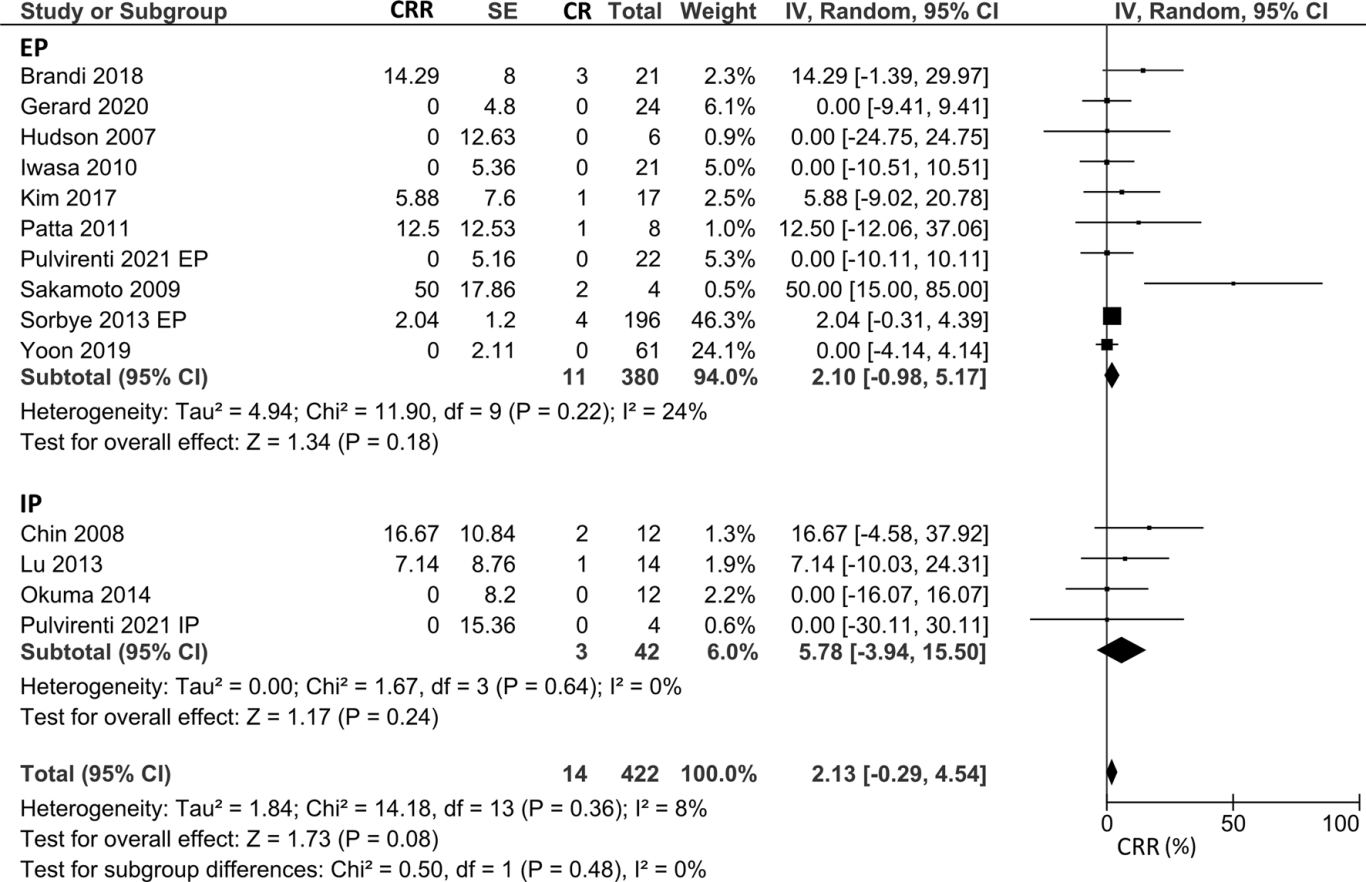
Pooled CRR in patients treated with platinum-doublet chemotherapy. 10 studies that evaluated patients treated with EP and four studies that evaluated patients treated with IP were included in this analysis. *CRR* complete response rate, *EP* platinum plus etoposide, *IP* platinum plus irinotecan

Regarding CRR, 10 studies with 391 participants evaluating EP and four studies with 45 patients assessing IP were included. The pooled CRR in all patients who received the platinum-doublet regimen was 2.1% (95% CI − 0.3% to 4.5%), and subgroup analysis for the EP and IP groups showed CRR of 2.1% (95% CI − 1.0% to 5.2%) and 5.8% (95% CI − 3.9% to 15.5%), respectively (Fig. 4). The CDDP/ETP and CBDCA/ETP groups showed CRRs of 1.6% (95% CI − 0.8% to 4.0%) and 12.0% (95% CI − 11.0% to 35.1%), respectively (Supplementary Fig. 2). Studies directly comparing the CRR of the EP regimen with that of the IP regimen were not available.

### Meta-analysis of survival time

**Fig. 5:**
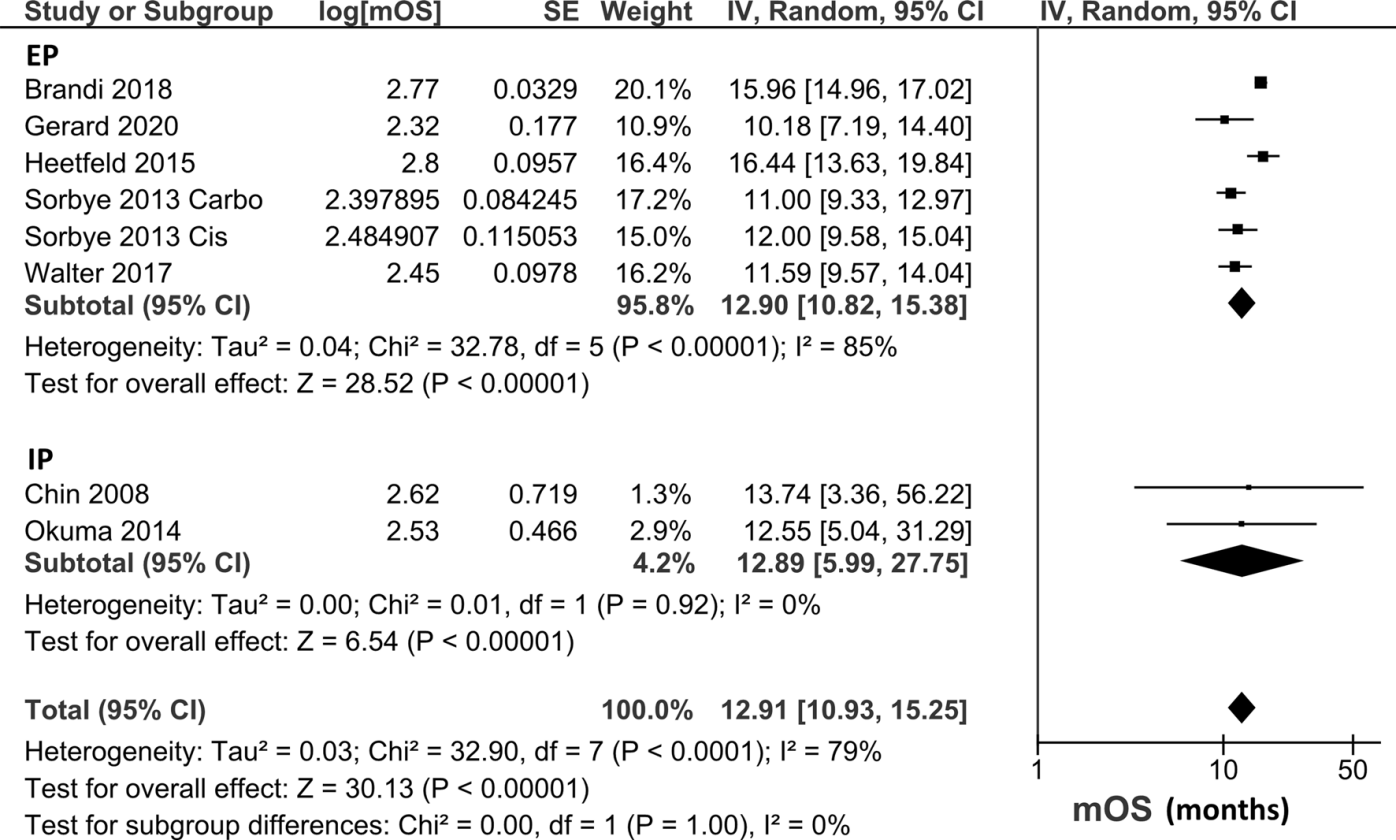
Pooled median OS in patients treated with platinum-doublet chemotherapy. Five studies that evaluated patients treated with EP and two studies that evaluated patients with IP were included in this analysis. *EP* platinum plus etoposide, *IP* platinum plus irinotecan, *OS* overall survival

**Fig. 6:**
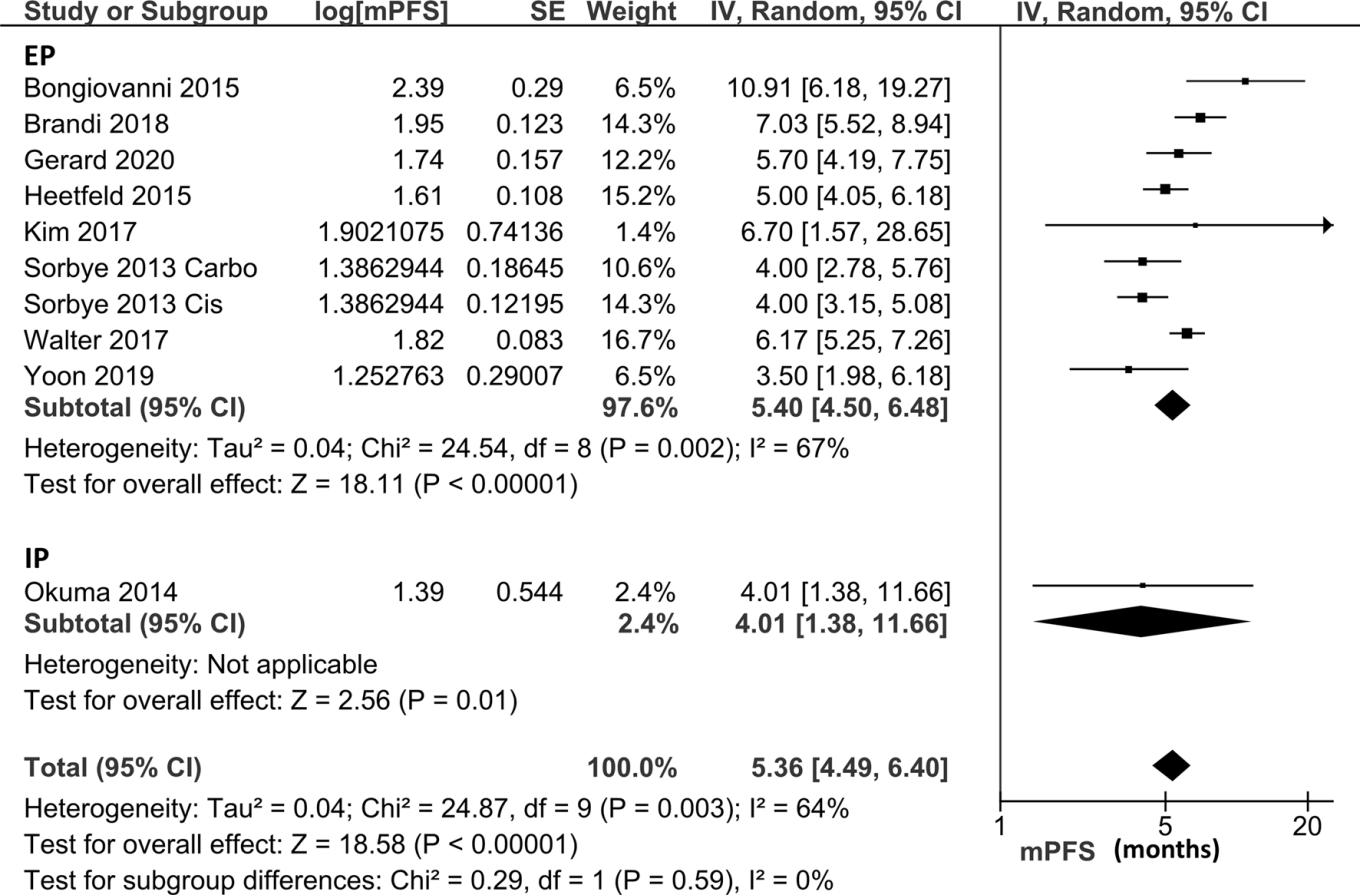
Pooled median PFS in patients treated with platinum-doublet chemotherapy. Eight studies that evaluated patients treated with EP and one study that evaluated patients with IP were included in this analysis. *EP* platinum plus etoposide, *IP* platinum plus irinotecan, *PFS* progression-free survival

Only one study that directly compared EP and IP documented the hazard ratio (HR) of OS. OS and PFS data at a specific time were insufficient in the included studies; therefore, median OS and PFS were integrated to assess the efficacy of the EP and IP regimens (Table 1). The pooled median OS in all patients who received the platinum-doublet regimen was 12.9 months (95% CI 10.9–15.3 months), whereas the EP and IP subgroups had the median OS of 12.9 months (95% CI 10.8–15.4 months) and 12.9 months (95% CI 6.0–27.8 months), respectively (Fig. 5). The Median OS of the subgroups based on each regimen is shown in Supplementary Fig. 3. The pooled median PFS was 5.4 months (95% CI 4.5–6.4 months) in all patients with platinum-regimen, 5.4 months (95% CI 4.5–6.5 months) in the EP subgroup, and 4.0 months (95% CI 1.4–11.7 months) in the IP subgroup (Fig. 6). The Median PFS of the subgroups based on each regimen is shown in Supplementary Fig. 4.

## Discussion

To the best of our knowledge, this is the first systematic review and meta-analysis of the clinical efficacy of platinum-doublet chemotherapy for advanced GEP-NEC. Most of the clinical data about this rare malignancy are derived from retrospective analyses, and only one randomized phase II trial data have been published to date [5]. Morizane et al. recently presented phase III trial (JCOG 1213) data between CDDP/ETP and CDDP/CPT-11 in 170 patients with GEP-NEC [24]. Our large-scale analysis, including more than 1100 patients, presents referential information about suitable chemotherapy options for GEP-NEC. According to the Surveillance, Epidemiology, and End Results database analysis including more than 160,000 NEC cases, the most common primary organ, except for the lung, was the GEP system, and the ratio of GEP-primary NEC to all extrapulmonary NEC cases was 37% [25]. In our analysis, studies that included extrapulmonary NEC other than GEP-NEC (e.g., urological or gynecological NEC) were excluded. Current treatment strategies generally differ between tumors in the GEP system and those in genitourinary or gynecological systems, and no uniform guidelines exist for NEC all over the body.

In our analysis, the pooled ORR in all patients with the platinum-doublet regimen was 49%, and subgroup analysis for the EP and IP groups exhibited ORRs of 44% and 59%, respectively. Three comparative studies between EP and IP did not detect significant differences in the ORR. These pooled median OS and PFS were similar between the two groups (OS: 12.9 months for EP and 12.9 months for IP; PFS: 5.4 months for EP and 4.0 months for IP). Although our analysis showed a favorable pooled ORR in EP and IP, the median PFS and OS of both treatments were short. In the JCOG 1213 trial, no significant difference was observed in OS or PFS between the EP and IP groups (median OS 12.5 months vs. 10.9 months; HR of EP to IP 1.04, 90% CI 0.79–1.37; median PFS 5.6 months vs. 5.1 months; HR 1.06, 95% CI 0.78–1.45). The ORRs were 54.5% and 52.5% in the EP and IP groups, respectively. These outcomes were generally consistent with those of our analysis. Several trials have demonstrated OS prolongation by adding anti-programmed death ligand 1 antibodies (atezolizumab or durvalumab) to EP, and a similar approach may be promising for GEP-NEC [26, 27]. The European Neuroendocrine Tumor Society guidelines state that CDDP/ETP is a standard regimen for GEP-NEC, and CPT-11 could be an alternative to ETP [28]. According to the European Society of Medical Oncology clinical practice guidelines for the treatment of advanced GEP-NENs, CDDP or CBDCA plus ETP is regarded as a standard first-line regimen for NEC [29]. Although the number of comparative studies between the regimens is limited, our results suggest no definite priority between EP and IP. Another interesting finding is the seemingly high ORR of CBDCA/ETP (67.2%) compared to that of CDDP/ETP (31.0%). It should be noted that the sample size in the CBDCA/ETP group was less than 100 in only three studies, and no comparative data were available between the two regimens. Therefore, we cannot make a definite comment on the priority of CBDCA/ETP over CDDP/ETP. However, CBDCA might be a better choice when patients are ineligible for CDDP due to the poor general condition or organ dysfunction. Our data support that CBDCA can be a reasonable substitute for CDDP.

For advanced SCLC, a direct comparison of CDDP plus ETP versus CDDP plus CPT-11 is available through several randomized clinical trials and meta-analyses. A phase III trial in Japan comparing CDDP/ETP and CDDP/CPT-11 demonstrated higher ORR and longer OS in patients treated with CDDP/CPT-11 (ORR 67.5%, 95% CI 55.9–77.8% vs. 84.4%, 95% CI 74.4–91.7%, *P* = 0.02; median OS 9.4 months vs. 12.8 months, *P* = 0.002; HR 0.60, 95% CI: 0.43–0.83) [30]. However, subsequent trials in the United States could not validate the superiority of CDDP/CPT-11 over CDDP/ETP for the ORR or OS [31, 32]. Two independent meta-analyses of randomized trials comparing the efficacy of EP and IP in patients with advanced SCLC showed an OS benefit in the IP group (Jiang et al., pooled HR 0.81, 95% CI: 0.66–0.99, *P* = 0.044; Lima et al. pooled HR 0.87, 95% CI: 0.78–0.97, *P* = 0.02) [33, 34]. The latest NCCN guidelines for SCLC refer to CDDP/ETP with durvalumab and CBDCA/ETP with atezolizumab or durvalumab as the preferred regimens [35]. The situation is more controversial in extrapulmonary NEC, including GEP-NEC.

Essentially, it is debatable whether chemotherapy response or prognosis is different between SCLC and extrapulmonary NEC. Terashima et al. retrospectively compared 95 SCLC cases and 41 extrapulmonary NEC cases, including 18 with gastrointestinal and 16 with hepatobiliary/pancreatic (HBP) NEC treated with a platinum-based regimen and reported that extrapulmonary NEC had a lower ORR than SCLC (78% vs. 31%, *P* < 0.01) [36]. Dasari et al. compared the prognosis of patients with extrapulmonary NEC to those with SCLC and reported that colon, pancreas, and liver NEC had worse OS (HR 1.09, 95% CI: 1.03–1.16; 1.10, 95% CI: 1.03–1.18; 1.85, 95% CI: 1.57–2.18) compared with SCLC, whereas small intestine NEC had better OS (HR 0.56, 95% CI: 0.49–0.64) than SCLC [25]. As a premise, the ORR in patients with SCLC from the aforementioned phase III clinical trials ranges from 44 to 68% in EP and from 48 to 84% in IP, and clinicians should consider such heterogeneous responses among studies comparing the efficacy of chemotherapy for SCLC and GEP-NEC [30–32].

Our study has several limitations. First, as one of the large faults, all other studies except for two studies included in this analysis were retrospective in nature. Second, safety analysis was not performed because of a lack of information in the studies included in this meta-analysis. Third, subgroup analysis between HBP-NEC and GI-NEC or SCLC and LCNEC could not be performed. As Yamaguchi et al. pointed out the distribution between HBP-NEC and GI-NEC affects the clinical efficacy of chemotherapy because clinical behaviors are originally worse in patients with HBP-NEC than in those with GI-NEC [6]. This limitation needs to be addressed by conducting a larger multi-institutional and international clinical trial or by performing an individual patient data meta-analysis. Fourth, the results of CRR obtained from our analysis were highly heterogenous partly due to a limited number of available studies. Therefore, caution should be applied when interpreting results. Finally, to simply assess the efficacy of chemotherapy in patients with GEP-NEC, our analysis excluded several studies that categorized patients with both GEP-NEC and non-GEP NEC as “patients with NEC.”

In conclusion, our novel systematic review and meta-analysis to assess the efficacy of platinum-doublet chemotherapy in patients with GEP-NEC showed considerable ORR. Subgroup analysis, including three comparative studies between EP and IP, did not detect any significant ORR differences. In terms of efficacy, there is no definite superiority between EP and IP, and both regimens are reasonably applicable to patients with GEP-NEC. We believe that these results provide reference information to clinicians for choosing suitable chemotherapy for this rare malignancy. Larger randomized clinical trials comparing these regimens and individual patient data meta-analyses integrating each patient’s clinical information are needed to stratify “good responders” to each treatment and identify predictive factors for each therapy, which provides robust clinical evidence for patients with GEP-NEC.

## Supplementary Information

Supplementary Figure 1. Forest plots of ORR in each platinum-containing regimen. Seven studies that evaluated patients treated with ETP/CDDP, three with ETP/CBDCA, six with ETP/platinum, six with CPT-11/CDDP, and one with CPT-11/platinum were included in this analysis. Platinum means cisplatin, carboplatin, or platinum anti-cancer agent not designated in the respective report. CBDCA, carboplatin; CDDP, cisplatin; CPT-11, irinotecan; ETP, etoposide; ORR, overall response rate. (TIF 937 KB)

Supplementary Figure 2. Forest plots of CRR in each platinum-containing regimen. Five studies that evaluated patients treated with ETP/CDDP, three with ETP/CBDCA, three with ETP/platinum, three with CPT-11/CDDP, and three with CPT-11/platinum were included. Platinum means cisplatin, carboplatin, or platinum anti-cancer agent not designated in the respective report. CBDCA, carboplatin; CDDP, cisplatin; CPT-11, irinotecan; CRR, complete response rate; ETP, etoposide. (TIF 803 KB)

Supplementary Figure 3. Forest plots of median OS in each platinum-containing regimen. One study that evaluated patients treated with ETP/CDDP, one with ETP/CBDCA, four with ETP/platinum, and two with CPT-11/CDDP were included. Platinum means cisplatin, carboplatin, or platinum anti-cancer agent not designated in the respective report. CBDCA, carboplatin; CDDP, cisplatin; CPT-11, irinotecan; ETP, etoposide; OS, overall survival. (TIF 480 KB)

Supplementary Figure 4. Forest plots of median PFS in each platinum-containing regimen. Three studies that evaluated patients treated with ETP/CDDP, one with ETP/CBDCA, five with ETP/platinum, and one with CPT-11/CDDP were included in this analysis. Platinum means cisplatin, carboplatin, or platinum anti-cancer agent not designated in the respective report. CBDCA, carboplatin; CDDP, cisplatin; CPT-11, irinotecan; ETP, etoposide; PFS, progression-free survival. (TIF 500 KB)

Supplementary file 5 (DOCX 299 KB)

## Data Availability

All data generated or analyzed during this study are included in this article. Further inquiries can be directed to the corresponding author.
