# Supplementary figures and images for: Platinum-doublet chemotherapy for advanced gastroenteropancreatic neuroendocrine carcinoma: a systematic review and meta-analysis
Source: Discov Oncol. 2022 May 30;13:40. doi: 10.1007/s12672-022-00499-w (PMC9151982; doi:10.1007/s12672-022-00499-w)

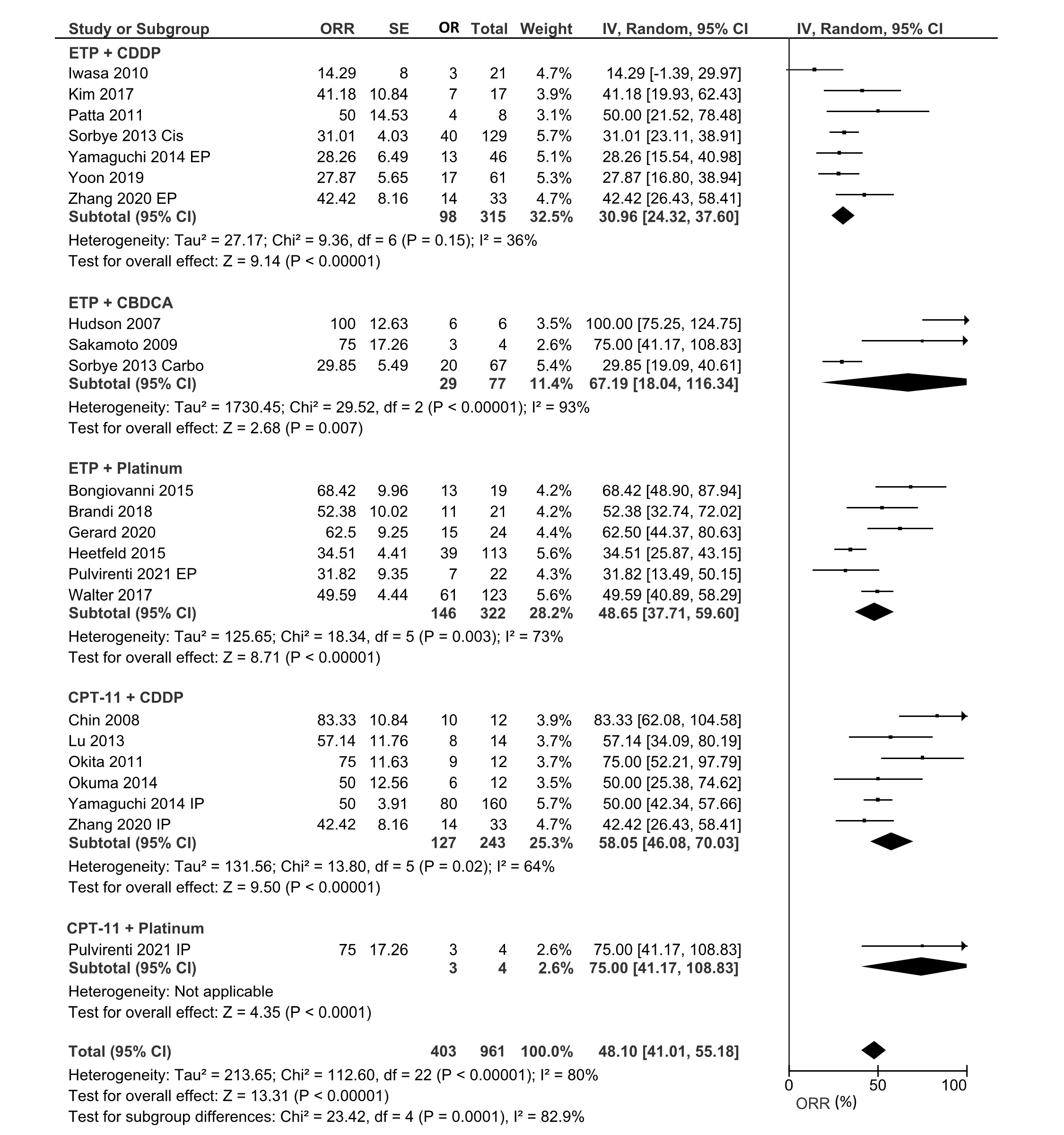

Supplement: Supplementary file 1 — Supplementary Figure 1. Forest plots of ORR in each platinum-containing regimen. Seven studies that evaluated patients treated with ETP/CDDP, three with ETP/CBDCA, six with ETP/platinum, six with CPT-11/CDDP, and one with CPT-11/platinum were included in this analysis. Platinum means cisplatin, carboplatin, or platinum anti-cancer agent not designated in the respective report. CBDCA, carboplatin; CDDP, cisplatin; CPT-11, irinotecan; ETP, etoposide; ORR, overall response rate. (TIF 937 KB) [file 12672_2022_499_MOESM1_ESM.tif]

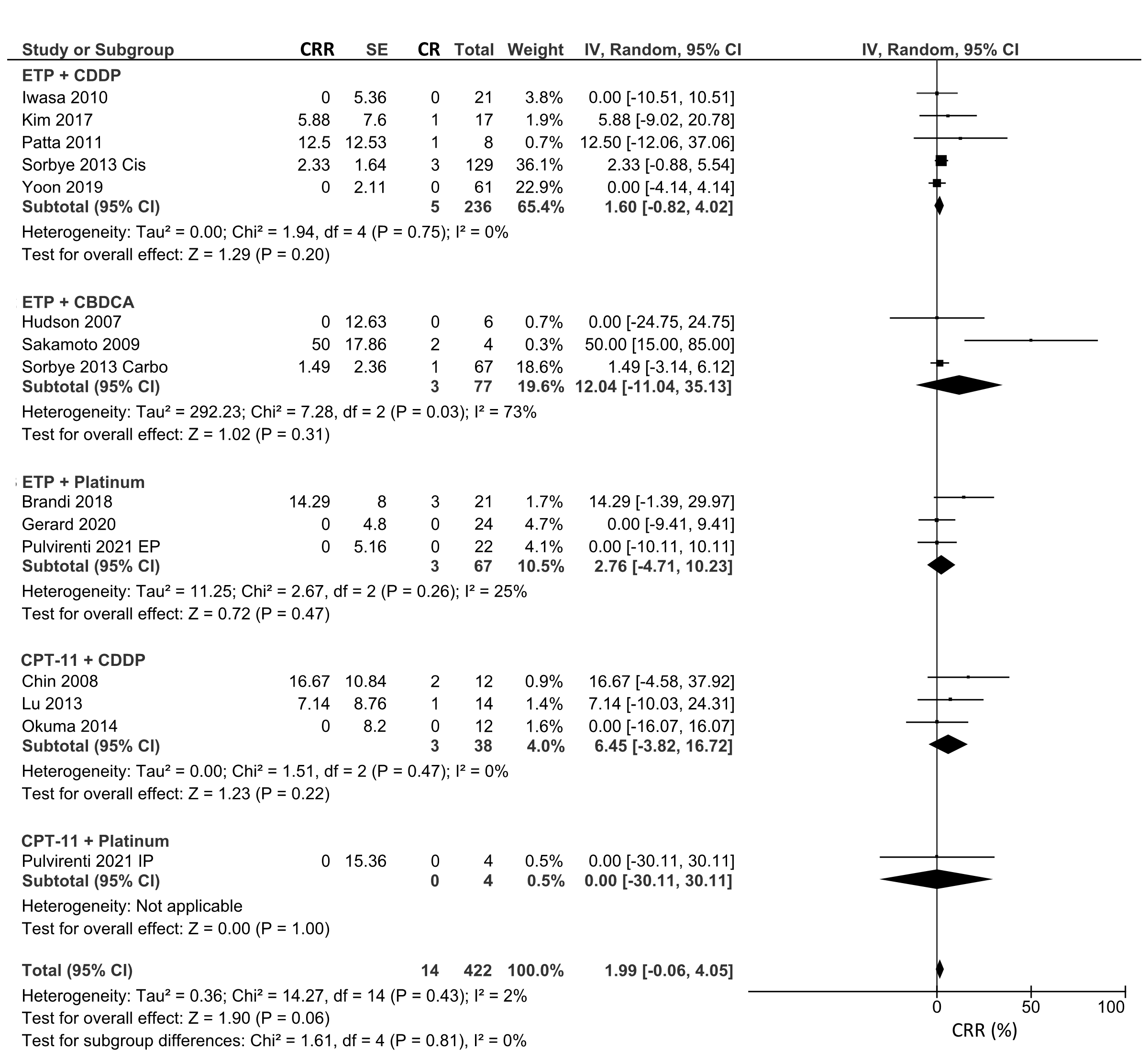

Supplement: Supplementary file 2 — Supplementary Figure 2. Forest plots of CRR in each platinum-containing regimen. Five studies that evaluated patients treated with ETP/CDDP, three with ETP/CBDCA, three with ETP/platinum, three with CPT-11/CDDP, and three with CPT-11/platinum were included. Platinum means cisplatin, carboplatin, or platinum anti-cancer agent not designated in the respective report. CBDCA, carboplatin; CDDP, cisplatin; CPT-11, irinotecan; CRR, complete response rate; ETP, etoposide. (TIF 803 KB) [file 12672_2022_499_MOESM2_ESM.tif]

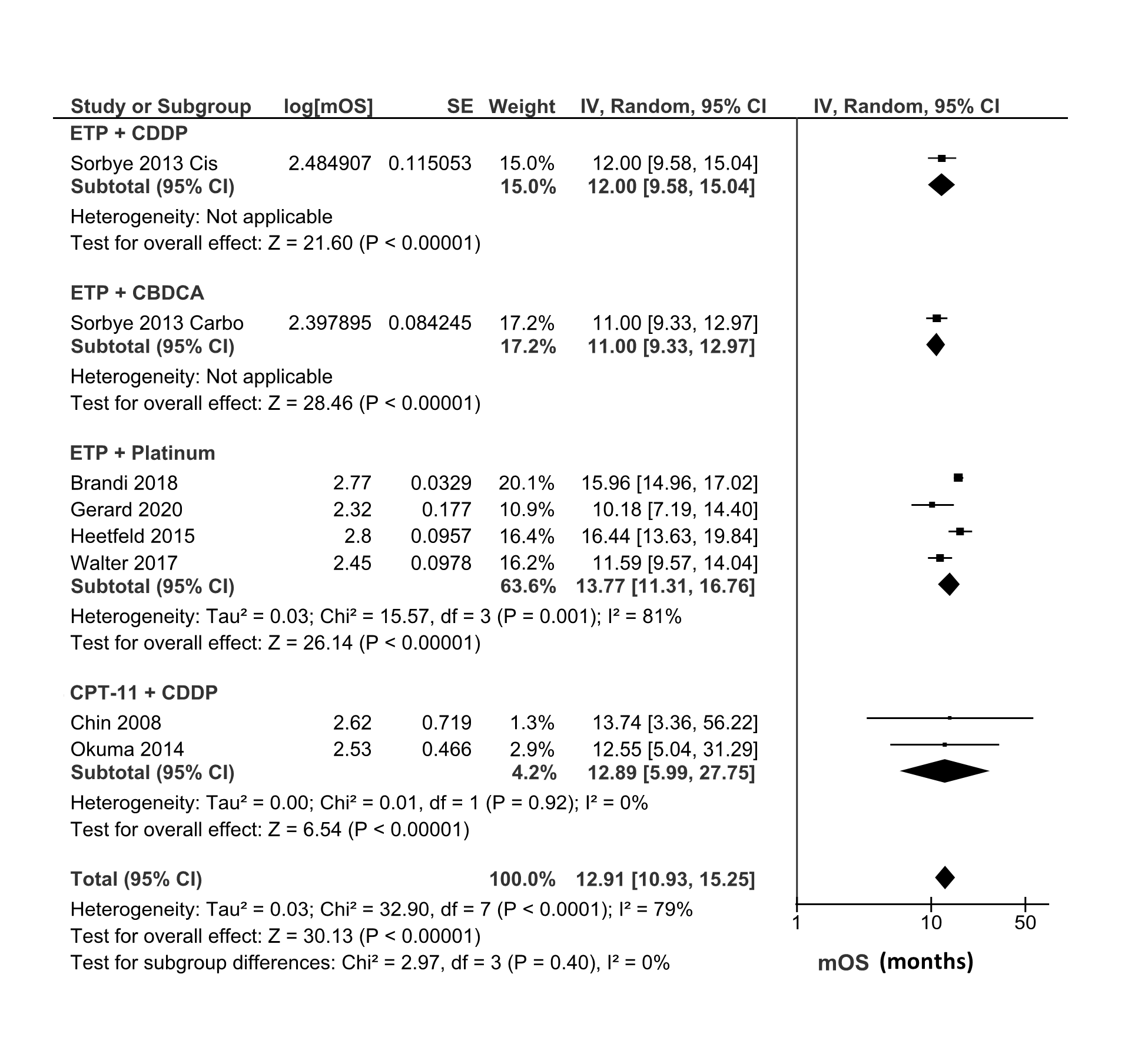

Supplement: Supplementary file 3 — Supplementary Figure 3. Forest plots of median OS in each platinum-containing regimen. One study that evaluated patients treated with ETP/CDDP, one with ETP/CBDCA, four with ETP/platinum, and two with CPT-11/CDDP were included. Platinum means cisplatin, carboplatin, or platinum anti-cancer agent not designated in the respective report. CBDCA, carboplatin; CDDP, cisplatin; CPT-11, irinotecan; ETP, etoposide; OS, overall survival. (TIF 480 KB) [file 12672_2022_499_MOESM3_ESM.tif]

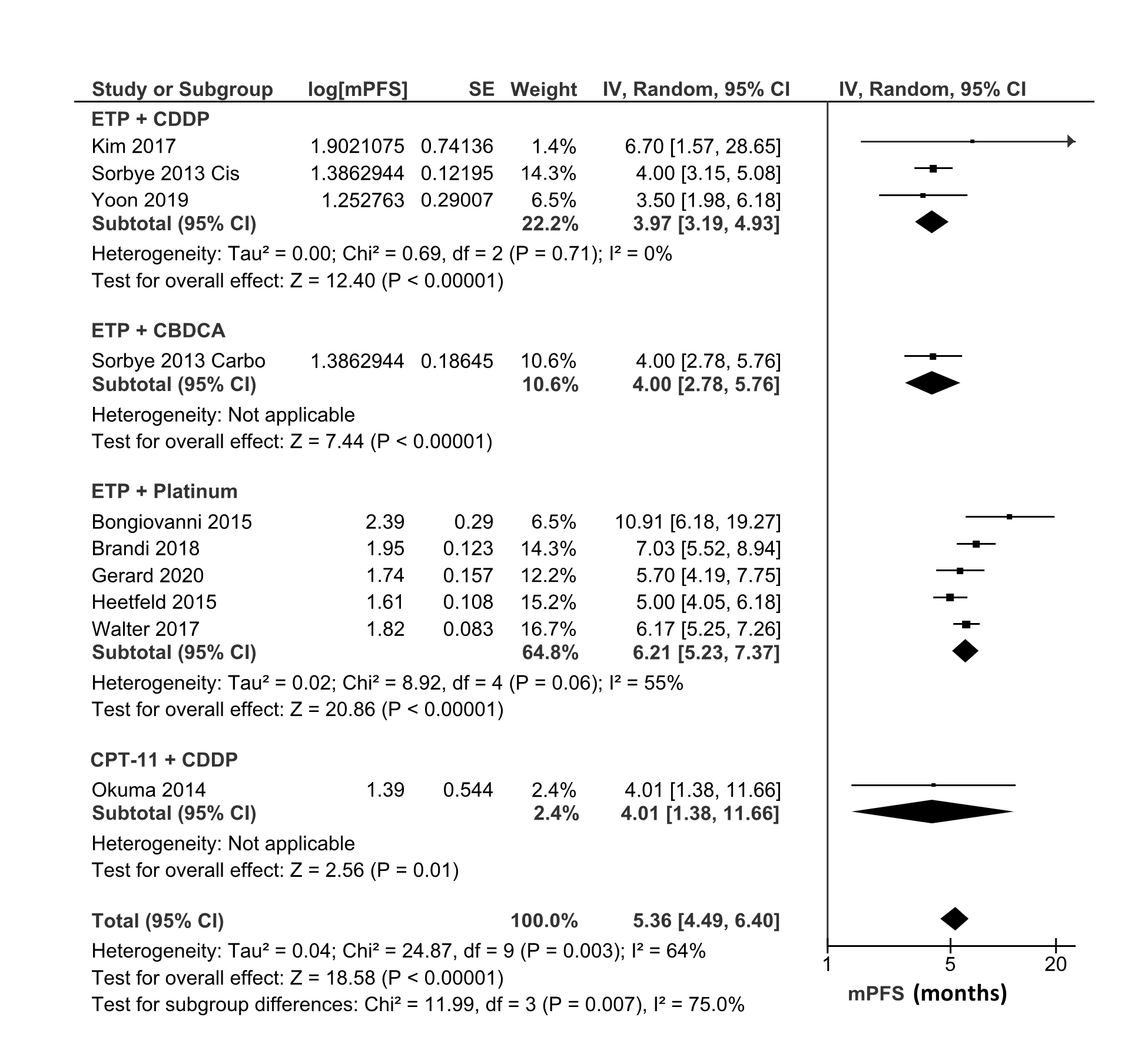

Supplement: Supplementary file 4 — Supplementary Figure 4. Forest plots of median PFS in each platinum-containing regimen. Three studies that evaluated patients treated with ETP/CDDP, one with ETP/CBDCA, five with ETP/platinum, and one with CPT-11/CDDP were included in this analysis. Platinum means cisplatin, carboplatin, or platinum anti-cancer agent not designated in the respective report. CBDCA, carboplatin; CDDP, cisplatin; CPT-11, irinotecan; ETP, etoposide; PFS, progression-free survival. (TIF 500 KB) [file 12672_2022_499_MOESM4_ESM.tif]
