# Supplementary material for: Platinum-doublet chemotherapy for advanced gastroenteropancreatic neuroendocrine carcinoma: a systematic review and meta-analysis
Source: Discov Oncol. 2022 May 30;13:40. doi: 10.1007/s12672-022-00499-w (PMC9151982; doi:10.1007/s12672-022-00499-w)
Supplement: Supplementary file 5 — (DOCX 299 KB) [file 12672_2022_499_MOESM5_ESM.docx]

Supplementary Table 1. Risk of bias evaluation of included studies

Supplemantary Table 1-1. Evaluation of randomized studies


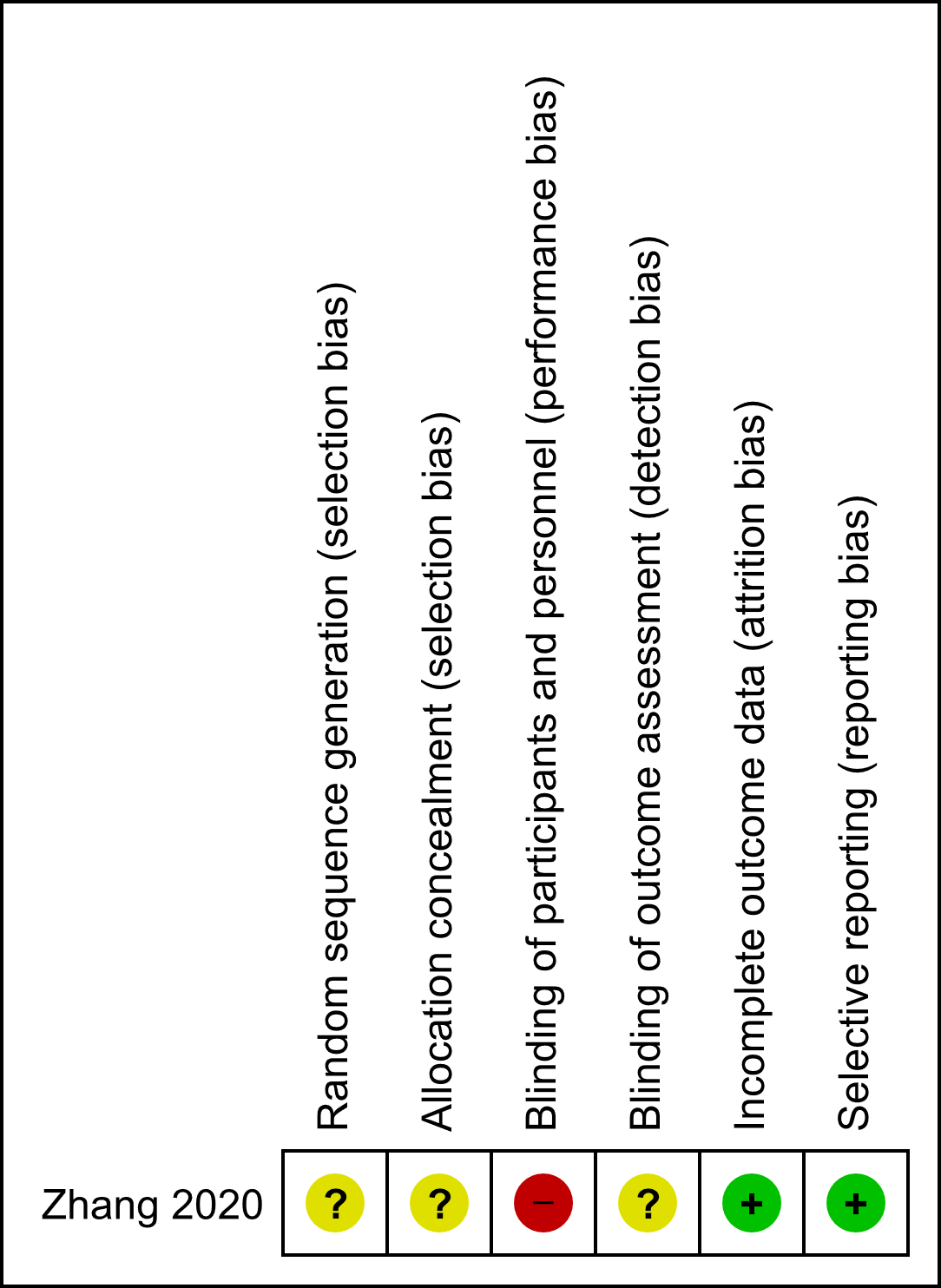


Only one study (Zhang et al, reference 6) was a randomized trial included in meta-analysis. This study was an open-label phase 2 study and ways of random sequence generation, allocation concealment, and blinding of outcome assessment were not documented in pulication and registry.

Green: Low risk of bias, Yellow: Unclear bias, Red: High risk of bias

Supplementary Table 1-2. Evaluation of non-randomized studies

| **Authors** | **Selection** |  |  |  | **Comparability** | **Outcome** |  |  |  |  |
| --- | --- | --- | --- | --- | --- | --- | --- | --- | --- | --- |
|  | Representative of the exposed cohort | Selection of the non-exposed cohort | Ascertainment of exposure | Outcome of interest not present at the start of the study |  | Assessment of outcomes | Sufficient follow-up time | Adequacy of follow-up | Total Score | Ref |
| Yamaguchi | * | - | * | * | - | * | * | * | 6 | 6 |
| Sorbye | * | - | * | * | - | * | * | * | 6 | 7 |
| Okuma | - | - | * | * | - | * | * | * | 5 | 8 |
| Lu | * | - | * | * | - | * | * | * | 6 | 9 |
| Iwasa | * | - | * | * | - | * | * | * | 6 | 10 |
| Okita | - | - | * | * | - | * | - | - | 3 | 11 |
| Chin | - | - | * | * | - | * | * | * | 5 | 12 |
| Yoon | * | - | * | * | - | * | * | * | 6 | 13 |
| Patta | * | - | * | * | - | * | * | * | 6 | 14 |
| Walter | * | - | * | * | - | * | * | * | 6 | 15 |
| Brandi | * | - | * | * | - | * | * | * | 6 | 16 |
| Heetfeld | * | - | * | * | - | * | * | * | 6 | 17 |
| Bongiovanni | * | - | * | * | - | * | * | * | 6 | 18 |
| Hudson | - | - | * | * | - | * | * | * | 5 | 19 |
| Sakamoto | - | - | * | * | - | * | * | - | 4 | 20 |
| Kim | * | - | * | * | - | * | * | * | 6 | 21 |
| Gerard | * | - | * | * | - | * | * | * | 6 | 22 |
| Pulvirenti | - | - | * | * | - | * | * | * | 5 | 23 |

9-star Ottawa Newcastle Scale was used to evaluate quality of non-randomized studies included in meta-analysis. Main factors of comparability are age and sex. Additional factor of comparability is primary site of neuroendocrine carcinoma.
